# Profiles of academic and cognitive abilities differ in younger and older children from diverse socioeconomic neighbourhoods

**DOI:** 10.1080/00049530.2024.2435318

**Published:** 2024-12-09

**Authors:** Frank D. Baughman, Sally A. Cook, Simone K. Treasure, Amy Morley, Evan Dauer, Darren Haywood

**Affiliations:** a Neurocognitive Developmental Laboratory, School of Population Health, Curtin University, Perth, WA, Australia; b Human Performance Research Centre, INSIGHT Research Institute, Faculty of Health, University of Technology Sydney, Sydney, New South Wales, Australia; c Department of Mental Health, St. Vincent’s Hospital Melbourne, Fitzroy, VIC, Australia; d Department of Psychiatry, Melbourne Medical School, Dentistry and Health Sciences, University of Melbourne, Parkville, VIC, Australia

**Keywords:** Socio-economic status, academic achievement, cognitive development and intelligence

## Abstract

**Objective:**

Research indicates that socioeconomic status (SES) influences developmental outcomes, particularly in language, executive functions, and intelligence, though findings have been mixed. This study examines the relationship between academic, cognitive and intellectual abilities in a cross-section of children at two age levels in low-SES vs. high-SES schools.

**Method:**

We administered a computerised battery of tests to 46 children in Grade Two (youngest 6.9 years old) and 67 children in Grade Six (oldest 12.4 years old) across four primary schools from low-SES and high-SES neighbourhoods. The test battery comprised two academic ability tests, five cognitive ability tests, and two intelligence tests.

**Results:**

In Grade Two, the low-SES group showed disadvantages on all measures except the Wisconsin Card Sorting Task and Choice Reaction Time. In Grade Six, while academic differences persisted between SES groups, cognitive differences were limited to the Wisconsin Card Sorting Task, where the high-SES group performed better than the low-SES group.

**Conclusions:**

Though our results pertain to cross-sectional data using neighbourhood indices of SES, our findings contrast with previous research showing broad and pervasive disadvantages associated with lower SES. Future research should further examine the potential differences and similarities in developmental outcomes across SES groups using longitudinal methods.

## KEY POINTS

**What is already known about this topic**:

1. Socioeconomic status (SES) influences developmental outcomes.
2. Research has highlighted broad disadvantages for children from lower SES backgrounds across cognitive and academic measures.
3. Mixed findings exist regarding the extent and specificity of SES-related developmental differences.

**What the topic adds**:

1. Children from low-SES schools demonstrated significant disadvantages in academic, cognitive, and intelligence measures in Grade Two, except on the Wisconsin Card Sorting Task and Choice Reaction Time.
2. By Grade Six, while academic differences between SES groups persisted, cognitive differences narrowed, with the high-SES group outperforming the low-SES group only on task (the Wisconsin Card Sorting Task).
3. These findings challenge assumptions of pervasive SES-related disadvantages and underscore the need for longitudinal research to understand developmental trajectories across SES groups.

Socioeconomic status (SES) is used across various contexts to differentiate individuals according to the relative amount of education, income, and type of job held. A large amount of literature shows that SES is associated with differences in the quantity and quality of resources individuals can access. SES has been associated with pre- and postnatal differences in nutrition, childhood obesity, exposure to stress, trauma, drug use, and differences in the levels of cognitive stimulation available to children. Within the psychological literature, a great deal of the interest in SES stems from its relation to various developmental outcomes. For instance, numerous studies have shown that SES accounts for a significant proportion of variance on educational, cognitive, intellectual, physical, and mental health measures (Farah, 2018; Haider et al., 2022; Lawson & Farah, 2017; Peng & Kievit, 2020; Poulain et al., 2020; Tucker-Drob & Bates, 2016).

In relation to academic outcomes, SES has been shown to predict absenteeism, school grades, and the probability of dropping out of high school (Sirin, 2005; von Stumm et al., 2020). However, the potential effects of SES on academic outcomes have also been found earlier in life. For example, O’Donnell et al. (1995) found that on entry to kindergarten, children from high and low-SES backgrounds differed reliably in their knowledge of the alphabet, and ability to count and write their names. Additionally, these early impacts of SES are visible within the cognitive domain. For example, Hackman, Farah and Meaney (2010) reported reliable differences on working memory and inhibition tasks between children as young as six months old who were from higher versus lower SES households (see also e.g., Lee & Burkam, 2002). Still, other studies have shown that SES reliably differentiates performance on tasks measuring language abilities in children as young as 18 months of age (Peng & Kievit, 2020), and later on tasks measuring intelligence (Haider et al., 2022; Larson et al., 2015; Tucker-Drob & Bates, 2016). Of further concern is the finding that the disadvantage between children from low-SES versus high-SES backgrounds grows larger during the course of development (von Stumm S et al., 2015). This has important implications for theoretical accounts of development and practical applications such as early intervention strategies for at-risk populations. Additional claims have been made that SES has direct and substantive effects on the development of neurocognitive processes (Lawson & Hook, 2014; Noble et al., 2007; von Stumm S et al., 2015). In line with this, a small number of studies have reported differences between low and high-SES groups in the development of temporal and frontal brain regions – areas assumed, respectively, to be crucial for language and regulating and controlling memory and attentional processes (Thomas et al., 2023).

Whilst there is now clear evidence showing SES is associated with important outcomes in development, significant challenges exist for approaches seeking to establish the functional associations relating to how factors interact across social, environmental, biological, and cognitive levels.^1^ Thus, questions remain as to exactly how SES affects the processes that are assumed to underlie academic, cognitive, and intellectual development. To further establish functional accounts of how SES relates to these outcomes and what interventional strategies may be applied, a strong understanding of the relative impacts of SES during early development is critical.

This study aims to contribute to our understanding of how SES affects developmental outcomes across childhood by investigating the relative differences in ability profiles between cohorts of younger and older children from primary schools in diverse SES neighbourhoods.

This study investigates the relative differences in ability profiles in cohorts of younger and older children from primary schools in diverse SES neighbourhoods. We take a cross-sectional approach to examine the extent to which these groups of children differ on tests assessing academic abilities, cognitive abilities and intelligence. Given existing findings, we expect to find (1) compared to children from lower SES neighbourhoods, children from higher SES neighbourhoods will show better performance on measures of academic ability, cognitive ability, and tests of intelligence, and (2) on all measures we expect to find larger relative differences between SES groups, in the older versus younger age groups.

## Method

### Design

Our study followed a quasi-experimental, cross-sectional design with two categorical, between-group independent variables. These were (1) Socioeconomic Status (low-SES vs. high-SES) and (2) School Grade (Grade Two vs. Grade Six). The dependent variables were performance scores (interval scale) on a battery of computerised tasks and pencil and paper measures (details of tasks are presented within the Measures and Apparatus subsection).

### Schools

We used census data compiled by the Australian Bureau of Statistics to identify schools of diverse (i.e., lower versus higher) SES neighbourhoods within the Perth metropolitan area, Western Australia. These data provide the Socio-Economic Indexes for Areas (SEIFA, 2011) that give estimates, in decile ranks with a range between 1 and 10, of the average socioeconomic characteristics of people, families, and households within a given postal area. Of the initial six school principals (three low-SES, three high-SES) that we contacted for the purposes of recruitment, four agreed to participate. Table 1 shows the socioeconomic indices for the four schools where testing was conducted. The indices are as follows: *Index of Relative Socio-economic Advantage and Disadvantage (IRSAD)*, *Index of Relative Socio-economic Disadvantage (IRSD)*, *Index of Economic Resources (IER)*, and *Index of Education and Occupation (IEO)*. Across the four indices of IRSAD, IRSD, IER and IEO, low scores indicate, respectively, greater disadvantage and lack of advantage,^2^ general disadvantage,^3^ lack of access to resources,^4^ and lower educational and occupational status.^5^ Table 1 also provides an Average Socio-Economic Index (ASEI) computed using the four indices, showing large overall differences between the SES of the three schools compared to the fourth school. These schools form our between-groups independent variable (low-SES vs. high-SES). At the time of testing, the low-SES school comprised 199 students, and the three high-SES schools had 344, 264 and 236 students, respectively.

**Table 1.** Summary table of socio-economic indices for areas of recruited schools.

	N	IRSAD	IRSD	IER	IEO	ASEI
*High-SES 1*	344	10	10	9	10	10
*High-SES 2*	264	10	9	7	10	9
*High-SES 3*	236	10	10	10	9	10
*Low-SES*	199	2	2	2	3	2

Data shown are of decile ranks for Indices of Relative Socio-economic Advantage and Disadvantage (IRSAD), Index of Relative Socio-economic Disadvantage (IRSD), Index of Economic Resources (IER), and Index of Education and Occupation (IEO). The computed average of decile ranks is given by the Averaged Socio-Economic Index (ASEI). *N* = the total number of children attending each school at the time of testing.

### Participants

Across the four schools from Grade Two and Grade Six, we obtained parental consent for 134 children with no known learning or developmental disorders. Data for 21 children were not included in the final data set due to missing data across the majority of tests (*N* = 19) and voluntary withdrawal (*N* = 2). The final sample consisted of 113 children (youngest 6.9 years, oldest 12.4 years old) from Grade Two (*N* = 46; mean age = 7.5 years, *SD* = 0.3) and Grade Six (*N* = 67; mean age = 11.4 years, *SD* = 0.4). Grade Two comprised 25 boys and 21 girls, and Grade Six comprised 41 boys and 26 girls.

### Measures and apparatus

The task battery comprised two tests of academic ability, five tests of cognitive ability, and two tests of intelligence. *Academic ability* was assessed using (1) Reading, measured via the Test of Word Reading Efficiency -second edition (Farah, 2018) and (2) Mathematical ability, measured via performance on subtraction, addition, multiplication and division problems. *Cognitive ability* was measured using (1) Balance Reasoning (e.g., Torgesen et al., 2012), (2) Visual Memory (e.g., Inhelder & Piaget, 1958), (3) Mental Rotation (e.g., Sperling, 1963), (4) Choice RT, and (5) WCST (e.g., Shepard & Metzler, 1971). *Intelligence* was assessed using (1) Raven’s standard progressive matrices (Heaton et al., 1993) and (2) an Inspection Time task (see, e.g., Raven, 1981). Except for Raven’s and TOWRE-2, administered in paper format, all other tasks were administered via standard Windows PCs supplied by schools. The computerised tasks were specifically designed for this study, as part of a larger research program aimed at evaluating children’s cognitive and non-cognitive abilities. Computerised tasks were developed using Java, and these were deployed to run locally on each machine, thereby providing millisecond-precision response times. Though the tasks were not normed against existing versions, pilot testing with diverse samples demonstrated they were sensitive measures for assessing children’s abilities across a range of proficiency levels – specifically avoiding ceiling or floor effects.

#### TOWRE-2

We used the Sight Word Efficiency subtest of the TOWRE-2 to assess children’s word reading accuracy and fluency. This test imposes a 45-second time limit and assesses one’s ability to read words presented on a single sheet of A4 paper. The dependent variable (DV) was the percentage of correctly identified words out of a maximum of 104 items. A wide range of previous research has shown the TOWRE-2 to be sensitive, valid, and reliable (e.g., see Marinus et al., 2013; Tarar et al., 2015).

#### Mathematics

Children completed four 2-minute computerised tests of addition, subtraction, multiplication, and division. Problems were presented in a fixed order, starting with relatively easy, single-digit problems (e.g., *“2 + 2 = ?”, “6–1 = ?”, “3 × 1 = ?”*, and *“4 ÷ 2 = ?”*), and these became progressively more challenging; involving 2 and 3-digit numbers (e.g., *“34 + 152 = ?”, “132–29 = ?”, “12 × 63 = ?”*, and *“232 ÷ 4 = ?”*). All problems were presented in white Verdana, font size 32, centred on-screen against a black background. Children used the numeric keypad on a keyboard to enter their answers to each problem. Feedback (“*Correct*!”/“*Wrong*”) was presented for 600 ms after each trial. The DV was the number of correct trials summed across problem type. Previous research has shown similar tests to be sensitive, valid, and reliable (e.g., see Nosworthy et al., 2013).

#### Balance Reasoning

A total of 96 balance scale problems were administered. These comprised 12 trials each of six problem types (balance, distance, weight, conflict-balance, conflict-distance, and conflict-weight). Children used the mouse to click an on-screen button to indicate whether they believed the scale would “tip left”, “balance”, or “tip right” after removing the supporting blocks. Feedback (“*Correct*!” / “*Wrong*”) was presented for 600 ms after each trial. The DV was the percentage correct out of the maximum 96 trials. Previous research in related populations has shown balance reasoning tests such as these to be sensitive, valid, and reliable (e.g., see van der Maas & Jansen, 2003).

#### Visual memory

Children completed 40 trials of a Letter Matrix task. Each trial began with a white fixation cross, font size 50, against a black background. This remained on-screen until the participant pressed the keyboard space bar to indicate they were ready, and the presentation of the letter matrix immediately followed this. The matrix consisted of two rows of four letters, capitalised, in white Sans Serif font, font size 80, against a black background. The matrix remained on screen for 1000 ms. The order of letters in the matrix was randomised, and trials excluded the presentation of vowels. Following the stimulus offset, a cursor appeared, and participants were required to type the letters seen previously in their correct positions in the matrix. Participants were instructed to give their “best guess” if they reported not being sure or not remembering. Feedback displaying the number of correctly recalled letters on each trial was presented for 1000 ms. The DV was the percentage of letters correctly identified across all 40 trials. Letter matrix tasks have been highly utilised and have been shown to be sensitive, valid, and reliable (e.g., see Gur et al., 2012).

#### Mental rotation

We administered 84 trials on a mental rotation task in which single characters (R, L, or F) were presented on screen in white Sans Serif font, font size 80, against a black background. These appeared either in their correct form (normal) or reverse (mirror) at one of seven possible orientations (0, 30, 60, 90, 120, 125, 180 degrees). Trials were counterbalanced so that each stimulus was presented six times at each orientation for normal and reverse presentations. Each trial began with a fixation cross in the middle of a blank screen. This remained on screen until participants pressed the space bar to indicate they were ready. Immediately following this, a blank screen was presented for 300 ms and a stimulus letter then followed. Participants were required to press either the left mouse-button if the stimulus image was a normal presentation (and potentially rotated) or the right mouse-button if the image was a mirror presentation (and potentially rotated). The DV, overall accuracy, was obtained by summing the number of correct trials for normal and reverse collapsed across each degree of orientation. Mental rotation tasks are some of the most highly used cognitive measures within this population and have been shown to be sensitive, valid, and reliable (e.g., see Jansen et al., 2013).

#### Choice RT

Children were administered 50 trials in which images of a white coloured arrow (400 × 300 pixels) were presented on screen against a black background. The order of presentation was randomised, with 25 trials showing the arrow facing left and 25 trials with the arrow facing right. Stimuli stayed on-screen for 1200 ms, after which, if a response was not made, the trial was recorded as incorrect, and the next item was presented. On-screen feedback provided participants with their response time (in milliseconds) for correct responses or the text “Wrong” if their response was incorrect. The DV was the average response time (in milliseconds) for correct trials. The choice relation time task is highly utilised within the literature with similar populations and has been shown to be sensitive, valid, and reliable (e.g., see Robbins, 2002).

#### Wisconsin Card Sorting Task (WCST)

Children were administered a total of six blocks for which they were required to match a card according to a single dimension corresponding to either its colour, shape, or number. The underlying dimension-to-match-to was not given. Children used the PC mouse to click and drag the card to one of four piles. On-screen feedback (“*Correct*!” / “*Wrong*”) enabled children to learn the correct dimension-to-match-to. Once participants had correctly identified the strategy, and once they provided correct responses to 10 consecutive trials, the dimension-to-match-to was changed. Blocks were randomised in their presentation however no two consecutive blocks were alike. The DV was the percentage of correct trials, given the number of attempts, across all 6 blocks.^6^ The WCST is highly utilised within cognitive research in related populations and has been shown to be sensitive, valid, and reliable (e.g., see Romine et al., 2004).

#### Raven’s standard progressive matrices

Children were administered the standard sets (A-E) from Raven’s standard progressive matrices (Anderson, 1988) in groups. This followed the procedure detailed in the testing manual, with children completing as many of the 60 problems as possible within 20 minutes. The DV was the percentage of correct trials out of 60. The Raven’s standard progressive matrices has been shown to be highly sensitive, valid and reliable in school-aged children (e.g., see Pind et al., 2003).

#### Inspection time

A total of 96 trials were administered in which a white fixation cross, font size 50, appeared against a black background. This remained on-screen until the participant pressed the keyboard space bar to indicate they were ready, and this was immediately followed by a trial showing an image of an alien bug. Images were 200 × 200pixels in size and presented for a maximum of 1000 ms. Participants were required only to indicate by clicking one of two on-screen buttons whether the antennae (or feelers) of the alien bug had been identical (“*Same”*) or different (“*Different”*) in length. A simple staircase algorithm tracked accuracy on each trial, reducing exposure durations by 25% following a correct response or increasing exposure duration by 50 ms following an incorrect response. Same/Different trials appeared with equal frequency but were randomised in their presentation. The DV was the shortest exposure duration (in milliseconds) for which responses were correct 75% of the time. The inspection time task has been found to be highly sensitive, valid and reliable in school-aged children (e.g., see Anderson, 1988).

### Procedure

Following ethical approval from the Department of Education and Training and the host university (HR2022/23), the parents of each child at both Grade Two and Grade Six at each school were sent letters informing them of the study. Testing began following the return of written consent and after receiving students’ assent. All testing was conducted during school hours, in the schools’ libraries and in class times normally scheduled for reading or computer literacy lessons.

The battery of tasks was embedded within a group, game-like format in which children were instructed to work as quickly and accurately as possible. Each task began with the children sitting quietly to watch a short video demonstrating what they would be required to do. Several practice trials were demonstrated for each task to ensure children understood the task requirements before starting. School staff assisted in ensuring that each workstation’s monitor, keyboard, and mouse were set up to be at an appropriate distance and reach for each child. The full set of tasks was counterbalanced across three to four 30-minute sessions to reduce possible effects of fatigue and order. The total average time to complete all tasks was approximately 90 minutes.

## Statistical analyses

We used descriptive and parametric tests to examine the study’s hypotheses. Key performance metrics, including mean reaction time and percentage correct (accuracy), were analysed as indicators of relationships between socioeconomic status (SES) and academic, cognitive, and intellectual ability. Multivariate analyses of variance (MANOVA), in line with best practice guidance provided by Warne (2014), were used to test for significant differences between the groups across each dependent variable. The results are presented in two stages: first for younger participants (Grade 2), then older participants (Grade 6).^7^ Finally, we combined these findings to illustrate differences in ability profiles across developmental stages. All statistical analyses were conducted using IBM SPSS Statistics (Version 29) with an alpha level of *p* < 0.05.

## Results

### Hypothesis 1

**Figure 1.**
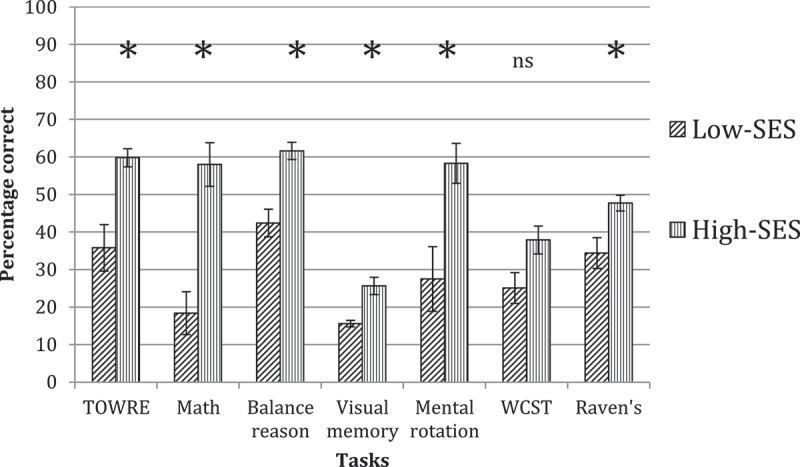
Accuracy on tasks in low-SES versus high-SES groups at grade two. Asterisks indicate reliable group differences; ‘ns’ indicates non-reliable group differences.

**Figure 2.**
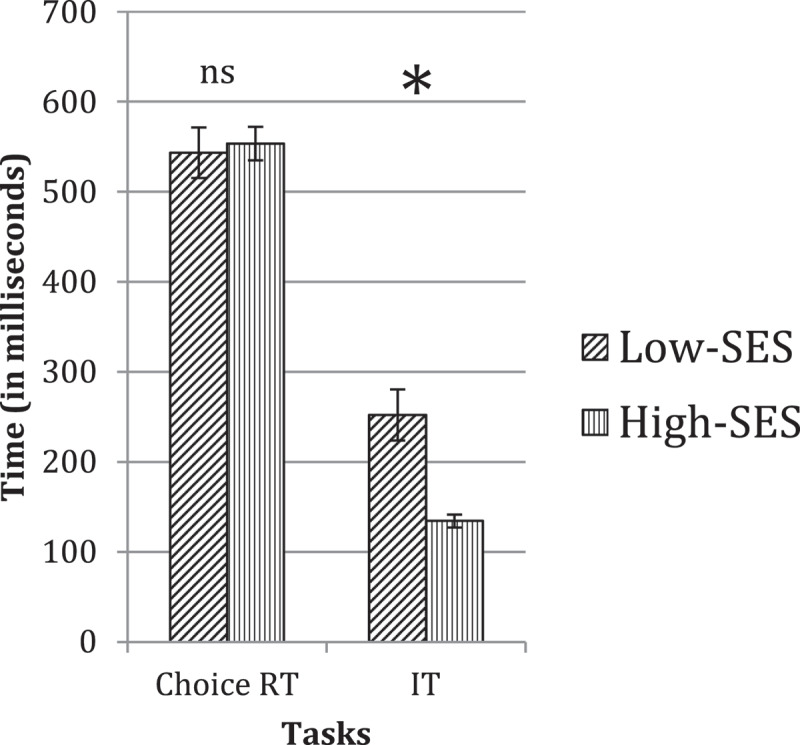
Speed of processing in low-SES versus high-SES groups at grade two. Asterisks indicate reliable group differences; ‘ns’ indicates non-reliable group differences.

Comparisons between the SES groups at Grade Two showed that the high-SES children outperformed the low-SES children on all but one of the tasks we administered (the exception was the Choice RT task, where the low-SES group showed better performance). The relative profiles for both groups on tasks assessing accuracy versus tasks assessing processing speed can be seen in Figure 1 and Figure 2, respectively. A MANOVA confirmed that the differences between groups were reliable on: TOWRE-2 (*F*(1, 40) = 18.88, *p* < 0.001, η^2^ = .321), Math (*F*(1, 33) = 14.04, *p* = .001, η^2^ = .312), Balance Scale (*F*(1, 31) = 21.90, *p* < .001, η^2^ = .414), Visual memory (*F*(1, 23) = 10.23, *p* = .004, η^2^ = .308), Mental rotation (*F*(1, 34) = 10.52, *p* = .003, η^2^ = .236), and Raven’s (*F*(1, 39) = 10.31, *p* = .003, η^2^ = .209). Only one non-reliable group difference was observed on the WCST (*F*(1, 32) = 1.99, *p* = .168). These results show evidence of clear differences between the low and high-SES groups in their academic, cognitive and intellectual abilities.

Figure 2 shows the overall group performance on the two speed of processing tasks we used. While the Low-SES group showed a slight advantage over the high-SES group in their faster overall responses on the Choice RT task, this difference was not reliable (*F*(1, 40) = 0.09, *p =* .760). On the IT task, it was the high-SES group that once again showed reliably better performance compared to the low-SES group (*F*(1, 28) = 16.25, *p* < .001, η^2^ = .367). These results suggest equivalent motor abilities in the two groups but faster information-processing rates in the high-SES group.

**Figure 3.**
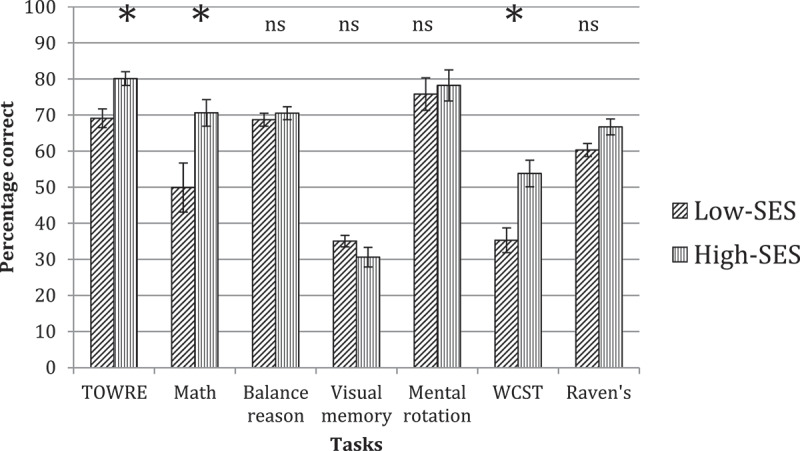
Accuracy on tasks in low-ses versus high-ses groups at grade six. Asterisks indicate reliable group differences; ‘ns’ indicates non-reliable group differences.

**Figure 4.**
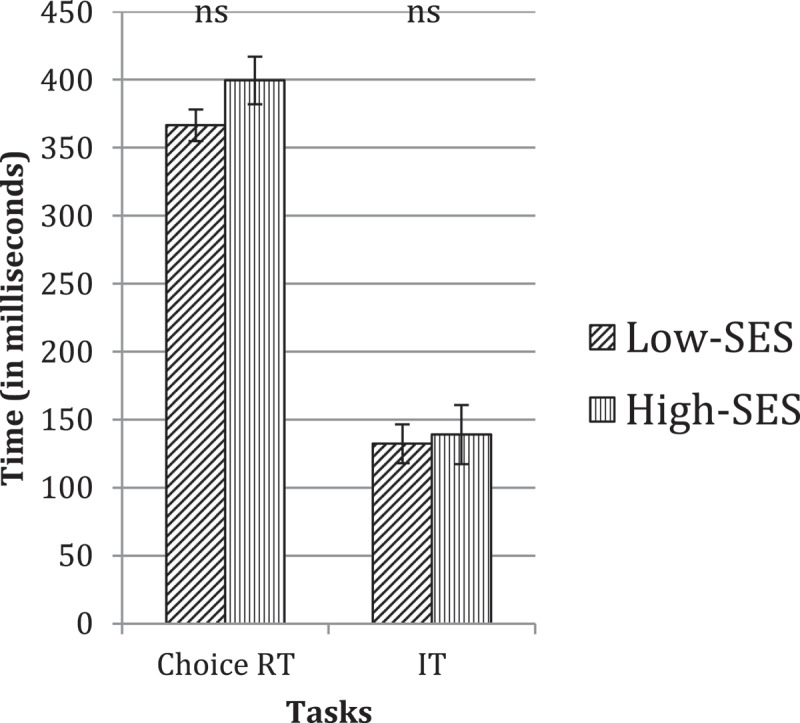
Speed of processing for low-ses versus high-ses groups at grade six. ‘ns’ indicates non-reliable group differences.

In contrast to the results obtained for the younger age group, the ability profiles obtained at Grade Six show fewer differences between SES groups (see Figure 3 and Figure 4). The results of a MANOVA on these data revealed reliable group differences on only three of the nine tasks. These were on TOWRE-2 (*F*(1, 38) = 9.74, *p* = .003, η^2^ = .204), Math (*F*(1, 53) = 8.48, *p* = .005, η^2^ = .128) and WCST (*F*(1, 56) = 11.99, *p* = .001, η^2^ = .176). On each of these tasks, it was the high-SES group who showed overall better performance compared to the low-SES group. The small advantage of the low-SES group over the high-SES group on the Visual Memory task was not reliable (*F*(1, 43) = 1.25, *p =* .269). None of the group differences were reliable on Balance (*F*(1, 60) = 0.47, *p =* .494), Mental Rotation (*F*(1, 43) = 0.12, *p =* .726), or Raven’s (*F*(1, 43) = 3.91, *p =* .052).

On the two tasks measuring speed of processing, the low-SES group showed marginally better performance compared to the high-SES group (see Figure 4). However, neither of these group differences was found to be reliable (Choice RT: *F*(1, 38) = 2.70, *p* = 0.109; IT: *F*(1, 49) = 0.06, *p* = .805), indicating that at this age the low and high-SES groups are equivalent in their motor abilities and their rate of information-processing.

### Hypothesis 2

**Figure 5.**
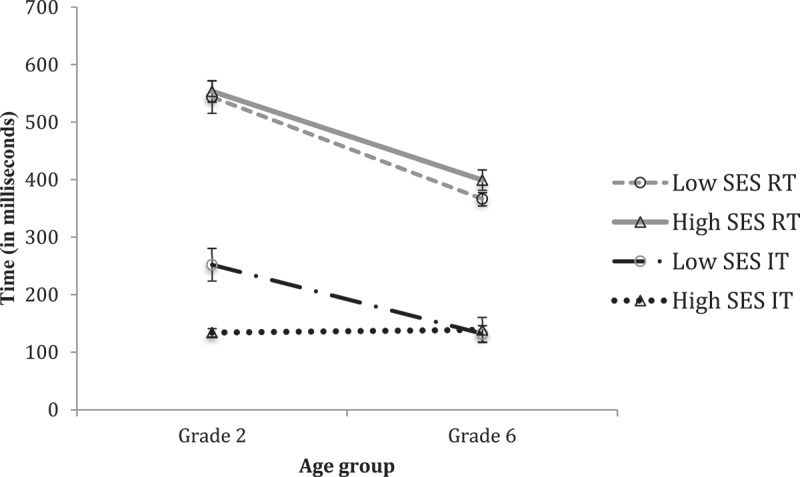
Relative differences in motor (RT) and speed of processing (IT) in grade two and grade six, in low-ses versus high-ses groups.

Next, we present results combining Grade with SES on tasks measuring academic, cognitive, and intellectual abilities. While Box’s M test indicated heterogeneity of variance-covariance matrices (F = 3.792, *p* < .010), we proceeded with MANOVA as this analysis is robust to such violations when sample sizes are adequate and violations are not extreme (Tabachnick & Fidell, 2019). To ensure conservative interpretations, we adopted a more stringent alpha level of .025 for subsequent comparisons. Although these data are cross-sectional, analysing the results in this way enables a comparison of the ability profiles of SES groups in younger and older groups. Figure 5 shows reaction time and speed of processing, measured by the Choice RT and IT tasks, of Grade 2 and Grade 6 children in the low-SES and high-SES groups. Consistent with existing developmental evidence of increased motor control with age, MANOVA revealed a reliable main effect of Grade on RT performance (*F*(1, 64) = 89.293, *p* < .001, η^2^ = .582; no Grade by SES interaction was observed). With regard to performance on the IT task, the analysis revealed a reliable main effect of Grade (*F*(1, 64) = 20.137, *p* < .001, η^2^ = .239) and a reliable Grade by SES interaction (*F*(1, 64) = 6.671, *p* = .012, η^2^ = .094). As Figure 5 shows, whereas the high-SES group shows little difference in IT performance in the two age groups we tested, the older low-SES group appears considerably improved, and now equivalent to the older high-SES children, in their ability to process information.

**Figure 6.**
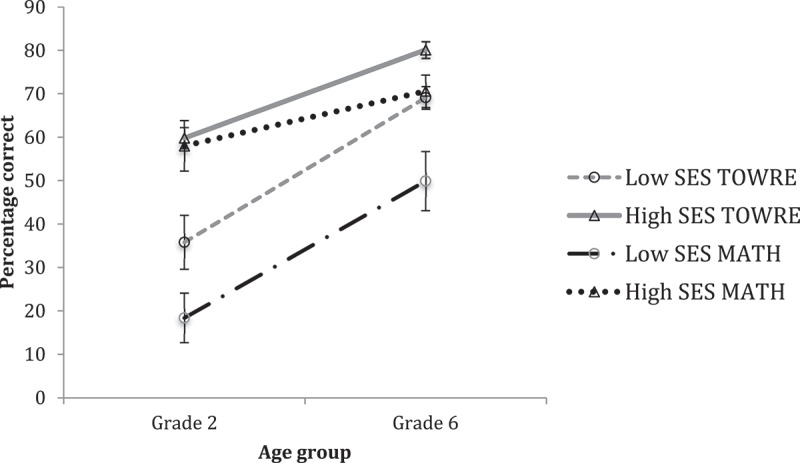
Relative differences in academic ability in grade two and grade six, in low-SES and High-ses groups.

Academic performance for children at Grade 2 and Grade 6 for each SES group is shown in Figure 6. MANOVA revealed reliable main effects of Grade on TOWRE-2 (*F*(1, 62) = 70.470, *p* < .001, η^2^ = .532), and Math (*F*(1, 62) = 10.356, *p* = .002, η^2^ = .143), and reliable main effects of SES on TOWRE-2 (*F*(1, 62) = 33.374, *p* < .001, η^2^ = .350) and Math (*F*(1, 62) = 21.994, *p* < .001, η^2^ = .262). These results fit with previous findings showing a broad disadvantage in lower SES groups. However, the analysis also revealed a reliable Grade by SES interaction on TOWRE-2 (*F*(1, 62) = 9.007, *p* = .004, η^2^ = .127). As Figure 6 shows, this was not due to a growing discrepancy but rather a reduced difference in abilities of SES groups at this older age. No reliable Grade by SES interaction was observed on Math (*F*(1, 62) = 3.184, *p* = .079, η^2^ = .049).

**Figure 7.**
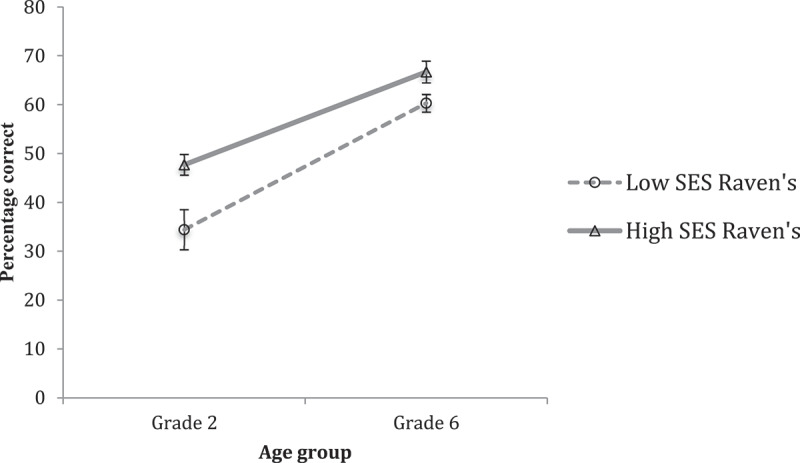
Relative differences in intellectual ability in grade two and grade six, in low-ses and high-ses groups.

Performance on Raven’s matrices task is shown in Figure 7. Results of a MANOVA showed reliable main effects of Grade (*F*(1, 104) = 72.729, *p* < .001, η^2^ = .412) and SES (*F*(1, 104) = 14.100, *p* < .001, η^2^ = .119). However, once more, and in contrast to expectations of a growing discrepancy between SES groups, the analyses showed no Grade by SES interaction. Indeed, Figure 7 indicates the differences between SES groups on this task are reduced at Grade Six.

**Figure 8.**
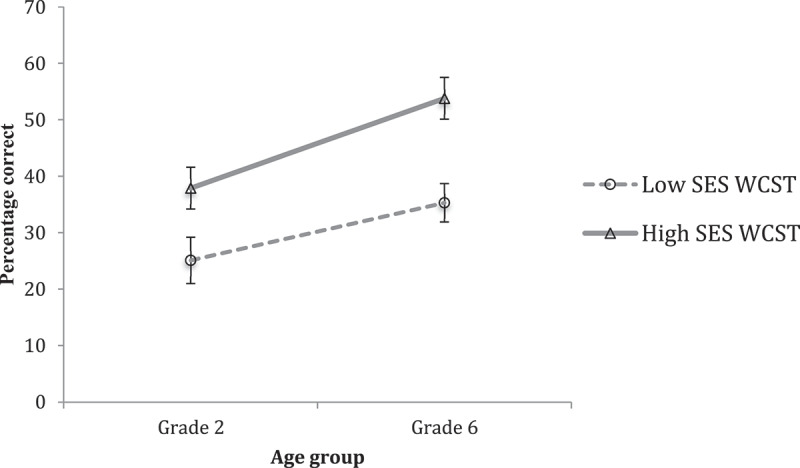
Relative differences on the WCST task in grade two and grade six, in low-SES and High-ses groups.

Performance on the WCST at Grade Two and Grade Six for both SES groups is presented in Figure 8 . The results of a MANOVA on these data revealed a main effect of Grade (*F*(1, 88) = 5.841, *p* = .018, η^2^ = .062) and SES (*F*(1, 88) = 8.410, *p* = .005, η^2^ = .087). Though for the first time, Figure 8 appears to show signs of a growing discrepancy between SES groups at Grade 6 versus Grade 2, this difference in profiles was not reliable (no reliable Grade by SES interaction was obtained).

## Discussion

The aim of this study was to investigate the relative differences in ability profiles in cohorts of younger and older children from primary schools in diverse SES neighbourhoods. In comparing the performance profiles at Grade Two, our results showed clear differences in children’s academic, cognitive, and intellectual abilities in high-SES versus low-SES groups. For example, on the academic (school-based) reading and mathematics tests, we found reliable group differences favouring children in the high-SES group. Group differences were also evident on the cognitive tasks we used. Overall, the high-SES group outperformed the low-SES group on the balance scale task, mental rotation, visual memory, and the WCST. These differences were reliable on all but the WCST. On the two measures of intelligence, group differences were found between low-SES and high-SES groups. Here, the high-SES group demonstrated greater scores on Raven’s, and faster speed of information processing, as measured by the IT task. Taken together, these findings are consistent with the literature that lower SES confers disadvantages for children early on in their development (e.g., Korous et al., 2022; Poulain et al., 2020; Raven, 1981).

The results of our analyses of groups at Grade Six, however, showed markedly different profiles associated with SES. Though clear group differences remained at Grade Six on measures of academic ability (i.e., high-SES showed superior performance on the reading and mathematics tasks), the groups differed reliably on just one of the cognitive tasks we used. This difference, on the WCST, indicates greater reactive cognitive flexibility in the high-SES group. Specifically, these results suggest that the older high-SES children were better able to adapt and switch between rules or categories in response to changing information. Lastly, whereas in the younger age group we found reliable group differences on both measures of intelligence, we obtained no such group differences at Grade Six. In contrast to the results relating to Grade Two, the analyses of profiles at Grade Six do not offer similar evidence of disadvantage associated with SES.

In looking to the differences in ability profiles observed between SES groups at Grade Two and then at Grade Six, several findings should be highlighted. First, our results are largely in line with the view that SES is associated with a disadvantage in academic ability (e.g., von Stumm S et al., 2015). However, the relative differences between younger and older cohorts at Grade Six were not congruent with research showing a growing SES-related disadvantage. Instead of finding evidence of an increasing gap between Grade Two and Grade Six in low versus high-SES groups, we found the gap in academic abilities to be *decreased*. This pattern of a narrowing discrepancy was also observed across the other measures we used, except for the WCST – this was the only task for which group performances showed a widening of discrepancies in abilities at Grade Six in low versus high-SES groups. These results may reflect a greater range of dynamic influences on the development of children in the lower SES groups. At least within this age range, the increased frequency of structured educational experiences that the lower SES children receive on starting primary school may help to promote changes in development, particularly in their general thinking skills (Baughman & Anderson, 2021; von Stumm et al., 2020). Thus, it would be of interest to determine what profiles would be found at other time points, later in childhood and adolescence.

One other result that stands out relates to the unevenness of academic versus cognitive and intellectual profiles of the low-SES group at Grade Six. While the performances of the low-SES at Grade Two are consistent with the idea that poorer IQ underlies poorer academic performance (e.g., Anderson et al., 2001), by Grade Six the low and high-SES groups were shown to be equivalent in their intellectual and cognitive abilities (except on the WCST). Despite this equivalence, however, the academic disadvantage in the low-SES group persisted. What might explain the relatively high cognitive abilities mixed with poorer academic skills? Whilst schools may make additional efforts to help the development of core competencies underlying cognitive and intellectual development, it is possible that the poorer academic performance of the low-SES group is affected by protracted development within social and emotional domains. This notion is supported by studies showing associations between lower SES, poorer adaptive functioning, and increased risk of mental health disorders (see e.g., Tucker-Drob & Bates, 2016). It is likely that these factors further interfere with children’s academic learning.

The finding that effects of socioeconomic status (SES) on cognitive processes appear to diminish with age and primarily impact school-related learning rather than basic cognitive abilities has significant implications for understanding the complex relationships between SES, education, and development. While our study provides initial evidence supporting this trend in primary school children, further research is needed to confirm whether these findings persist into secondary school years and beyond. A follow-up study using a sample of secondary school participants would be particularly valuable in examining how SES influences different aspects of education, such as academic achievement, motivation, or social-emotional development. Moreover, longitudinal data could provide insights into the temporal dynamics of SES-related differences by tracking students’ progress over time and examining whether these effects persist, change, or perhaps even reverse course during adolescence and young adulthood. Existing research suggests that factors such as self-discipline (Duckworth & Seligman, 2005) could play important roles in adolescence and beyond.

One limitation of our approach relates to the fact that we used a cross-sectional as opposed to a longitudinal design. Therefore, it is possible that the lack of SES group differences we observed at Grade Six reflected specific cohort effects. This might be due to targeted interventions aimed at helping children at risk. For example, research has shown positive effects of a range of physical and mental health interventions on academic achievement, school attendance and conduct, particularly for students from lower SES backgrounds (see e.g., Pickett & Wilkinson, 2015). Future studies adopting a longitudinal design aimed at replicating the results of the present work would help clarify this issue. A second limitation of our approach is that we did not collect per-child SES but instead used neighbourhood indices. Having these data for each child would offer greater sensitivity in allowing us to explore the potential effect of SES on the latent factors that may underlie academic, cognitive and intellectual abilities.

In summary, and notwithstanding these limitations, our findings offer a different view of the influence of SES on developmental outcomes. Our findings indicate that once children attend school, SES appears to exert its effects more on academic performance and less on the development of intellectual and cognitive abilities. That is, our findings appear to show that children’s academic, cognitive, and intellectual development are somewhat separable and that the influence of SES on these domains over development is not equal. Further, in contrast to the view that poor cognitive ability underlies poor academic ability, we show evidence of dissociation – the low-SES children were equivalent to the high-SES children on all but one cognitive measure. Thus, at Grade Six the low-SES children seem to be failing school-based academic measures for reasons other than their cognitive and intellectual ability (Farrington & Welsh, 2008). This finding highlights a critical issue in education, underscoring the need to move beyond simplistic explanations of academic underachievement. This is a significant finding with far-reaching implications for education policy and practice. By acknowledging the complex interplay of factors contributing to academic underachievement, educators can address the barriers that may prevent some students from reaching their full potential. For example, lower SES children may face additional challenges such as limited access to resources (e.g., technology, books), reduced parental support due to work commitments or other responsibilities, and increased exposure to stressors like poverty and housing insecurity – all of which can have a profound impact on academic performance. By recognising more of the possible contextual factors, educators can develop targeted interventions that address the root causes of underachievement.

The present study focused on exploring the relationship between children’s cognitive and intellectual abilities and their academic performance. The uneven profiles we found between younger and older children of lower versus higher SES suggest that the effects of lower SES may be modulated by other factors. Consequently, a more comprehensive understanding of the complex role that socioeconomic status plays in influencing children’s outcomes may be achieved by future work that incorporates the role of children’s social and emotional competencies (see e.g., Haider et al., 2022). Such research would not only deepen our understanding of how SES shapes educational outcomes but also inform targeted interventions aimed at promoting equity in education and mitigating potential disparities that arise from socioeconomic disadvantage (e.g., Paulus et al., 2021).

## Data Availability

Data may be made available at reasonable request of the corresponding author and to the satisfaction of the granting ethics committee.

## Notes

1. Though for a recent attempt at the development of such an approach, using data derived from a computational model of English past tense verb learning see Thomas et al. (2023).

2. Areas in which the majority of households are of low income, or majority of people are in unskilled occupations and few households are of high incomes, or few people are in skilled occupations.

3. The majority of households are of low income and there is a majority of people with no qualifications or a majority of people in low skilled occupations.

4. Areas in which the majority of households are of low income, or majority of households pay low rent and few households are of high income, or few owned homes.

5. The majority of people are without qualifications, or the majority of people are in low skilled occupations or the majority of people are unemployed and there are few with a high level of qualifications or in highly skilled occupations.

6. The literature shows a variety of approaches are taken in the scoring of performance on the WCST, and issues to do with finding suitable metrics reflecting EF processes are discussed at length (Barceló & Knight, 2002; Miles et al., 2021; Rosales-Lagarde & Martínez-Alcalá, 2021). In line with previous work (e.g., Haywood & Baughman, 2021), We opted to use measures of accuracy over e.g., perseveration errors on the WCST for two reasons. 1) Accuracy reflects a lack of general errors as well as lack of perseveration errors (after a category change). 2) we aimed to develop a comprehensive picture of children’s abilities (i.e., their ability to perform tasks correctly). Using accuracy on the WCST thus allowed us to maintain consistency in our results across all task measures.

7. By performing between-groups MANOVAs for each age group separately (low-SES vs high-SES), we were able to examine whether SES differences exist within a specific developmental context (i.e., at younger or older ages). This allowed us to test hypotheses about how SES affects performance in children of different ages, without the added complexity of interactions between variables.

## Highlights

- Socioeconomic status (SES) is associated with a broad range of developmental, educational and health outcomes.
- We used a battery of measures to assess the relationship between academic, cognitive and intellectual abilities in a cross-section of children in low-SES and high-SES school neighbourhoods at two age levels.
- At the younger age level, the low-SES group showed poorer performance on nearly all measures we administered. However, this pervasive disadvantage was not found in the older age group.
- Our findings indicate that greater discrepancies across multiple domains do not necessarily occur as a function of SES and that the differences in profiles may be modulated by a range of other factors e.g., children’s emotional and social abilities.
